# Association between coping of the primary caregiver and the adolescent patient with cancer

**DOI:** 10.1186/s12875-025-02965-0

**Published:** 2025-09-24

**Authors:** Jaramillo Villanueva Leonel, Mario Enrique Rendón Macías, Ana Ríos Covian

**Affiliations:** 1 Department of Mental Health, High Specialty Medical Unit, XXI Century National Medical Center Pediatric Hospital, Mexican Institute of Social Security, Av Cuauhtémoc # 330, Colonia Doctores, Alcaldía Cuauhtémoc, CP 06720 Mexico City, Mexico; 2 School of Medicine, Dental and Health Sciences Programs, National Autonomous University of Mexico, Panamerican University, Augusto Rodin # 498, Colonia Insurgentes Mixcoac, Alcaldía Benito Juárez, CP 03920 Mexico City, Mexico; 3 Department of Pediatric Oncology of the High Specialty Medical Unit, XXI Century National Medical Center Pediatric Hospital, Mexican Institute of Social Security, Av Cuauhtémoc # 330, Colonia Doctores, Alcaldía Cuauhtémoc, CP 06720 Mexico City, Mexico

**Keywords:** Psycho-oncology, Styles, Strategies, Coping, Adolescents, Cancer, Stress

## Abstract

**Background:**

Coping mechanisms help individuals face adversity, remain stable over time, and can be generalized to various circumstances. Two types are typically distinguished: the active style, aimed at resolving problems, and the passive style, focused on emotional regulation. We hypothesized that passive coping of the primary caregiver (hereafter, primary caregiver [PC]) would affect the adaptive coping of his or her adolescent child with cancer (hereafter, adolescent with cancer [AC]).

**Objective:**

To analyze coping styles in adolescents with cancer (ACs) and their primary caregivers (PCs).

**Materials and methods:**

This was an analytical cross-sectional study including 116 pairs of an adolescent with cancer (AC) and a primary caregiver (PC). The adolescents completed the Adolescent Coping Scale (ACS), applicable to those aged 9–17 years, while the caregivers completed the Coping Strategies Inventory (CSI).

**Results:**

49% (57/116) of the pairs both used the active coping style, and 14% showed the passive style in both members. No agreement was found between the coping styles of the AC and PC (Kappa = 0.15, 95% confidence interval [CI]: 0.13–0.14, *p* = 0.13). The multivariate analysis explained 61% of the variance (Nagelkerke pseudo R2 = 0.61; likelihood ratio = 191.4; *p* = 0.003).

**Conclusions:**

Passive coping by the primary caregiver occurred with low frequency, and active coping was favored, similar to that of the adolescent with cancer.

## Introduction

Cancer in adolescents represents the leading medical cause of death in developed countries1. Worldwide, more than 1 million new cases are diagnosed each year [1], including approximately 2,362 in Mexico, with a five‑year survival rate of 40–60% [2, 3]. To date, only a handful of studies have examined parent–adolescent dyadic coping in pediatric oncology, and virtually none have focused on Latin‑American settings, creating a critical evidence gap that limits culturally relevant clinical guidance. Against this backdrop, daily challenges for adolescents and families emerge.

An adolescent with cancer (AC) and his or her family face multiple stressors, such as diagnostic confirmation, repeated hospitalizations, separation from home, side effects of treatment, and uncertainty about potential relapses. These adolescents often experience anxiety, emotional or behavioral problems, and alterations in psychosexual development, body image, and independence, and sometimes develop psychological trauma [4–6]. These challenges set the stage for exploring how both members of the dyad cope with the illness.

For parents, a child’s oncological disease entails economic, social, physical, emotional, and occupational changes. Parental psychological well-being is strained by the fear for their child’s life [7]. Among the reactions commonly observed in the primary caregiver (PC) are bereavement, marital conflicts, overprotective parenting styles, and the transmission of fear-based beliefs, which may negatively affect the patient’s adaptation to treatment and subsequent psychological development [8, 9]. To understand how families manage these stresses, we turn to the Lazarus & Folkman’s coping framework.

According to Lazarus and Folkman, coping results from ‘cognitive and behavioral processes developed to manage external or internal demands that are evaluated as overflowing the resources of an individual’ [10]. This process regulates the negative consequences of stress by reducing and tolerating new demands [11]. In their conceptual framework, Lazarus and Folkman [10] distinguish two broad coping styles: an approach oriented toward problem-solving (often labeled active or adaptive) and one oriented toward emotional regulation (often labeled passive or maladaptive). However, both coping modalities can be advantageous depending on context [12]. Although active, problem‑focused strategies are generally associated with better psychological adjustment, emotion‑focused (passive) strategies may also be useful when direct action is impossible or personal resources are depleted [12]. Consequently, the most adaptive response often involves a flexible blend of problem‑ and emotion‑focused approaches tailored to the individual’s resources and the specific stressor [12].Yet few studies apply this framework to parent‑adolescent dyads in oncology.

Previous studies have primarily examined the emotional alterations and coping of adolescents diagnosed with cancer, often excluding the parents from analyses [13–16]. In our own clinical practice, however, we have repeatedly observed that some *primary caregivers* [PC] rely on passive, emotion‑focused strategies, prompting us to investigate how this style might influence their *adolescents with cancer* [AC]. We therefore hypothesised that passive coping in the PC would foster similarly passive coping in the AC and designed an analytical cross‑sectional study to test this assumption.

## Methods

A cross-sectional analytical study was carried out in a pediatric cancer center with adolescents aged 9 to 17 years over a six-month period. Each adolescent was invited to participate regardless of gender, along with the individual serving as the primary caregiver (defined as the family member responsible for the child’s care during cancer treatment). All adolescents had a confirmed oncological diagnosis and were undergoing treatment at any stage. Data collection followed immediately, using standardized questionnaires described below.

Questionnaires were self‑administered under the supervision of the principal investigator. Each adolescent completed the instruments at bedside without the caregiver present, and each caregiver did so separately, out of the patient’s view. Sociodemographic data were obtained at the same time through an ad‑hoc form filled out by the caregiver. The investigator explained the study, obtained written consent/assent, and remained available for questions.

### Adolescent Coping Scale (ACS)

Coping in adolescents was assessed with the Spanish-adapted, self-administered ACS. Previous Spanish validations report Cronbach’s α between 0.84–0.52 [17] while our results showed reliability between α = 0.83 and 0.72. In this study, problem-solving strategies were considered representative of active coping, whereas certain emotion-focused strategies corresponded to passive coping. Active coping was calculated as the sum of *problem solving*, *striving*, and *worrying* (M = 53, SD = 5, range = 6–60). Passive coping was the sum of *lack of coping*, *tension reduction*, and *self‑blame* (M = 31, SD = 5, range = 3–48). The criteria for determining coping styles were firstly identifying which coping style had the highest score and secondly verifying that no score lay outside the expected range (< 2 SD).

### Coping Strategies Inventory (CSI)

Primary‑caregiver coping was assessed with the Spanish version of the CSI. Earlier Hispanic validations report Cronbach’s α between 0.86–0.81, whereas internal results ranged from α = 0.76 to 0.67. Active coping was derived from the total of *problem solving*, *cognitive restructuring*, *social support*, and *emotional expression (M* = *44, SD* = *5, range* = *8–80); scores* < *2 SD below the mean were considered altered.* Passive coping combined *problem avoidance*, *wishful thinking*, *social withdrawal*, and *self‑criticism* (M = 26, SD = 4.5, range = 5–78); scores < 2 SD below the median were taken as out of range. These categories follow the problem‑ versus emotion‑focused typology of Lazarus and Folkman (1984) and have been validated in adolescent samples using the ACS and the CSI [10, 11, 18].

### Construct validity

A Varimax-rotated exploratory factor analysis confirmed the expected active-versus-passive structure in both scales (ACS eigenvalues > 1; KMO = 0.814, Bartlett *P* < 0.001; CSI KMO = 0.651, Bartlett *P* < 0.001).

#### Psychiatric assessment

Psychiatric diagnoses were established through a clinical evaluation by a child psychiatrist from the mental health service.

To measure family functioning, the Adaptability, Partnership, Growth, Affection, and Resolve (APGAR) questionnaire was employed [19], which in its Spanish validation showed a Cronbach’s alpha of 0.84 [20, 21]. Scores below 7 indicated family dysfunction.

#### Sample size

We estimated the sample size using the formula for proportions, assuming that 80% of patients would exhibit active coping, with a 95% confidence level, a 0.05 margin of error, and 20% potential dropout. This produced a target of 110 pairs [22].

#### Statistical analysis

Bivariate analysis was conducted using the chi-square test to examine differences in proportions of covariates and types of coping. Cohen’s kappa index was used to determine agreement between the coping styles of the adolescent and the primary caregiver. To identify factors related to passive coping in both members of the pair, we performed a multinomial logistic regression model, using the active–active coping pattern as the reference group. Factors are summarized as odds ratios (OR) with their respective 95% confidence intervals (CI). For data processing, we used SPSS version 23, and statistical significance was set at *p* < 0.05.

We ensured the anonymity and confidentiality of participants and provided psychological or psychiatric support as needed. *(Note: The ethical approval statement is relocated to the Declarations section.)*

## Results

We evaluated all adolescents with cancer hospitalized during the 6-month study period. A total of 129 individuals initially met the preliminary criteria, and 116 of these, along with their primary caregivers, proceeded to full study participation. The complete participant flow is detailed in Table 1.

**Table 1:** Flow of participants

Adolescents hospitalized during the study period	129
Excluded (13):	
• Intellectual disability (adolescent)	3
• Declined participation	6
• Deceased before study completion	2
• Caregiver illiterate	1
• Incomplete questionnaires	1
Included in the final sample	**116**

A majority of the adolescents with cancer were male (53%), and their mean age was 13 years. Most (81%) had solid tumors, while the rest presented with leukemia. On average, the disease had progressed for 4 months, with a median of 5 previous hospitalizations. Thirty-eight percent reported undergoing at least 1 surgery, and 55% had some form of organ failure or complication at the time of evaluation (e.g., bleeding, sepsis, pain, electrolyte imbalance, malnutrition, or perioperative status).

Fifty-seven adolescents (49%) had a psychiatric diagnosis, including 21 (18%) adaptive disorders, 12 (10%) depressive disorders, 9 (8%) generalized anxiety disorders, 7 (6%) mixed anxiety and depression disorders, and 5 (4%). separation anxiety, 2 (2%) trypanophobia and 1 (1%) anticipatory vomiting. Seventy-five percent knew their own diagnosis.

In terms of primary caregivers (PC), 81% were mothers, with a median age of 38 years. All of them knew the adolescent’s diagnosis; however, 64% perceived it as having a poor prognosis, 19% interpreted it as low-risk, and 17% were uncertain. Sixty-nine percent of these pairs came from outside Mexico City, and 55% (64 families) had established support networks.

Table 2 below summarizes these characteristics in one place, highlighting the background information collected in addition to the ACS and CSI measures.

**Table 2:** Sociodemographic and psychosocial characteristics of adolescents with cancer and their primary caregivers (*N* = 116)

	Adolescent (*N* = 116)	Primary caregiver (*N* = 116)
A. Sociodemographic variables		
Age^a^ (years)	13 (9–16)	38 (25–57)
Sex, female^b^	55 (47%)	94 (81%)
Education, years^a^	7 (2–11)	9 (6–18)
Place of origin, non-local^b^	80 (69%)	80 (69%)
Religion, Catholic^b^	100 (86%)	100 (86%)
Employment status, unemployed^b^	N/A	60 (52%)
Cancer diagnosis^b^	Solid neoplasms: 99 (85%) Hematologic neoplasms: 17 (15%)	N/A
B. Psychosocial variables		
Clinical prognosis (life-threatening)^b^	116 (100%)	N/A
Perceived prognosis*	N/A	Poor 64% Low-risk 19% Uncertain 17%
Knows own/patient diagnosis	Yes 75%	116 (100%)
Support networks available*	N/A	55%
Psychiatric diagnosis present	57 (49%)	52 (45%)
Family function (APGAR < 7)	0%†	0%†

*N/A* Not applicable

^a^Median (range: minimum–maximum)

^b^n (%)

^*^Psychosocial data were collected with the ad-hoc form completed by the caregiver

^†^All dyads scored ≥ 7 on APGAR, indicating adequate family function

### Coping styles

As shown in Table 3, active coping predominated: 78 adolescents (67%) and 79 caregivers (68%) reported an active style, but both members displayed active coping in only 57 pairs (49%).

**Table 3:** Behavior of the type of coping between the Caregiver (PC) and the Adolescent with Cancer (AC)

	AC Active	AC Passive	Total
PC Active	57 (49%)	22 (19%)	79 (68%)
PC Passive	21 (18%)	16 (14%)	37 (32%)
Total	78 (67%)	38 (33%)	116 (100)

Kappa index 0.15 (95% confidence interval: 0.130 to 0.144), p = 0.137

Passive coping of the primary caregiver appeared in 37 pairs (32%); in 21 of those pairs (56.7%) the adolescent countered with an active style. Conversely, among 38 adolescents who used passive coping, 22 (58%) had active compensation by their primary caregiver. The least favourable pattern—both members using passive coping—was observed in 14% of the pairs. Overall agreement between caregiver and adolescent coping styles was low (Kappa index = 0.15, 95% CI 0.13 to 0.14, *p* = 0.13).

### Characteristics of the pairs according to their coping style

According to the four possible combinations of coping, the comparative characteristics between groups are shown in Table 4. Regarding the type of coping and demographic characteristics, we did not find statistically significant differences, although there was a higher proportion of caregivers without partners in the pairs that exhibited passive coping.

**Table 4:** Characteristics of the population according to the coping styles of the Adolescent (AC) and the Primary Caregiver (PC)

Variables	AC & PC Active (*n* = 57)	AC Active, PC Passive (*n* = 21)	AC Passive, PC Active (*n* = 22)	AC & PC Passive (*n* = 16)
Demographics				
Patient’s age in years*†*	13 (9–16)	14 (9–16)	13 (9–16)	12 (9–16)
Patient’s gender*				
Male	31 (54%)	12 (57%)	10 (45%)	8 (50%)
Female	26 (46%)	9 (43%)	12 (55%)	8 (40%)
Caregiver’s age in years^+^	38 (25–56)	39 (29–50)	38 (30–57)	37 (27–46)
Caregiver's marital status*				
No partner	9 (8%)	5 (4%)	7 (6%)	6 (5%)
With partner	48 (41%)	16 (14%)	15 (13%)	10 (9%)
Total number of children^+^	2 (1–6)	2 (1–6)	2 (1–4)	2 (1–5)
Clinical				
Months of progression*				
< 7 months	30(53%)	17 (81%)	15 (68%)	8 (50%)
7 or more	27(47%)	4 (19%)	7 (32%)	8 (50%)
Surgeries*				
Without surgeries	21(18%)	9 (42.8%)	5 (26.1%)	5 (31.3%)
With surgeries	36(31%)	12 (57.2%)	17 (73.9%)	11 (68.7%)
Complications**				
Yes	38(67%)	6 (29%)	10 (45%)	10 (63%)
No	19(33%)	15 (71%)	12 (55%)	6 (37%)
Psychosocial				
Perceived prognosis*				
Poor	35 (61%)	12 (57%)	17 (77%)	10 (63%)
Well	12 (21%)	4 (19%)	1 (5%)	5 (31%)
Not defined	10 (18%)	5 (24%)	4 (18%)	1 (6%)
Knows his/her diagnosis**				
Knows	49 (86%)	13 (62%)	12 (55%)	13 (81%)
Does not know	8 (14%)	8 (38%)	10 (45%)	3 (19%)
Support networks*				
Has	36 (63%)	13 (62%)	7 (32%)	8 (50%)
Does not have	21 (37%)	8 (38%)	15 (68%)	8 (50%)
No. of hospitalizations***				
< than 6 Hospitalizations	33 (58%)	18 (86%)	17 (77%)	6 (37%)
≥ than 7 Hospitalizations	24 (42%)	3 (14%)	5 (23%)	10 (63%)
Psychiatric diagnosis PC*				
With	52 (45%)	14 (66.6%)	19 (90.4%)	14 (87.5%)
Without	5 (4%)	7 (33.4%)	3 (9.6%)	2 (12.5%)

^†^Kruskal–Wallis test p > 0.05

^*^Pearson Chi-squared test p > 0.05

^**^ *p* = < 0.05

^***^ *P* = 0.008

In relation to the clinical conditions, we found a higher proportion of patients with complications when the types of coping were shared as fully active or fully passive, compared to the “mixed” or compensatory coping scenarios (*p* = 0.017).

For psychosocial factors, the main difference between the types of coping was in the knowledge of the diagnosis by the adolescent; this was more frequent (> 80%) when there was either an active or passive agreement (*p* = 0.013). Additionally, agreement in passive coping was more common among those with multiple hospitalizations. Support networks were low (32%) in the group that combined passive coping in the adolescent and active coping in the caregiver, although this did not reach statistical significance. Family function (APGAR) was high in all groups, and there were no differences in psychiatric diagnoses across the groups.

### Multivariate analysis

Considering an active coping model in the pair (AC–PC) as optimal for managing the oncological process, we analyzed factors related to passive and compensatory coping styles.. The multinomial model, shown in Table 5, explained 61% of the variance. For the possibility of passive coping by both members, psychosocial factors such as having no partner (for the caregiver), younger caregiver age, and having more children emerged as most relevant. In this group, the adolescent was often unaware of his or her diagnosis and had multiple hospitalizations.

**Table 5:** Risk factors for coping style in the adolescent–caregiver pair (multinomial logistic regression)

Coping styles	Variables	OR	95% CI	*P*-value
Both passive	More than 6 hospitalizations	21.7	1.7—250	0.01
	Not knowing the diagnosis	11.5	0.9—132	0.05
	Having more children	10.9	1.1—109	0.04
	PC without partner	9.1	1.3—61.3	0.02
	PC age < 41 years	1.2	1.01—1.4	0.03
PC passive AC active	PC with psychiatric diagn	24.6	3.0—200	0.00
	Not knowing the diagnosis	9.0	1.5—55.5	0.01
	Patient < 12 years old	7.9	1.2—51.7	0.03
AC passive PC active	Not knowing the diagnosis	11.5	1.6—79.4	0.01
	Pair without support networks	7.0	1.4—34.8	0.01
	PC, perceive good prognosis	0.3	0.02—0.5	0.01
	Patient exposed to surgeries	7.1	1.00—100	0.05
Both active	Reference*			

Multinomial regression Pseudo R2 Nagelkerke 0.61.2% Likelihood ratio 191.4 *p* = 0.003. Adjusted for patient age (9–11 vs. 12–16), patient gender (male vs. female), age of primary caregiver (PC) 27 to 40 vs. 41 to 57, complications, psychiatric diagnosis of patient and psychiatric diagnosis of caregiver, and presence vs absence of family dysfunction

^*^In the multinomial logistic regression model (see Methods), “Both active” serves as the reference category because it represents the hypothesized optimal scenario where both adolescent (AC) and caregiver (PC) use active coping. All other coping-style combinations are compared against this baseline to determine how psychosocial and clinical factors shift the odds of adopting a different style

For the opposite compensation, with a caregiver with passive coping and an adolescent with active coping, the factors associated with the former were the presence of psychiatric illness and for the latter being a young adolescent and not knowing their diagnosis. In the compensatory situation of a passive style of the adolescent with an active coping of the caregiver, in the adolescents, it was associated with not knowing their diagnosis and having received some surgical procedure, in the primary caregiver it was perceiving the prognosis as favorable and especially if the family did not have an external support network.

## Discussion

### Clinical implications

The importance of studying coping in pediatric oncological diseases lies in the impact of these psychological mechanisms to manage fear and the uncertainty of treatment consequences, such as adaptation to repeated hospitalizations, medical procedures, and adverse news [6]. One key aspect often overlooked is the joint coping between parents and adolescents in the face of chronic illness. This process is inherently complex and involves emotional adaptation as well as modifications in family dynamics.22,23 Notably, in the literature search for our study, we did not find other reports describing precisely the same findings we observed regarding dyadic (parent–adolescent) coping styles in pediatric oncology.

A review of the scientific literature focusing on family coping strategies underscores that active coping strategies are associated with better adjustment and improved family functioning, whereas passive strategies may exacerbate stress or negatively affect family health [23]. In particular, Martínez-Montilla et al. (2017) highlight how families employing proactive, solution-focused approaches typically demonstrate greater resilience, while those with more avoidant or passive styles may face detrimental effects on emotional well-being. These observations reinforce our central premise—that assessing and addressing coping styles in both the adolescent and the caregiver is crucial for fostering a more supportive environment during cancer treatment.

In our study, the most important finding was that the passive coping style of the primary caregiver did not necessarily translate into a passive response in the adolescent; the concordance for passive coping between caregivers and adolescents was very low according to the Kappa index. Only fourteen percent of the pairs agreed on the passive coping style, which is similar to the withdrawal strategy findings reported by Sanjari et al. [12] when coping strategies were assessed individually. A second notable finding was that active coping in both members of the dyad emerged as the most likely scenario in the face of serious events, such as the adolescent’s cancer treatment. This occurred in approximately 50% of our cases. Finally, our results underscore the importance of always reviewing coping styles in each member of the dyad, given the low concordance in passive coping (Kappa = 0.15).

When pairs displayed active coping styles, did not typically represent a problem in cancer treatment, given their attitude toward adversity that combines cognitive efforts aimed at solving problems and controlling emotional responses [11, 18, 24]. By contrast, shared passive coping seemed to limit these efforts. However, we also identified situations of “compensatory” styles—observed in 37% of the sample—which proved favorable in some instances. In these cases, when at least one member showed a passive attitude, the other compensated with an active attitude.

This finding suggests that an unexpected lack of association between caregiver and adolescent coping could be explained by a phenomenon of compensatory coping: when one member of the pair exhibits a passive attitude, the other compensates by adopting an active stance, thereby creating a unified, adaptive front against the disease. A similar phenomenon is “mutual pretense,” where parents hide information about the disease to protect the adolescent from negative emotions, and the adolescent likewise remains silent to avoid distressing the parents [25]. It is also comparable to the resilience documented in children of depressed mothers or children of parents with cancer, where a close emotional bond can drive a “rescue” response to shield the other from fear or sadness [26, 27]. In this regard, we observed that in the absence of an active coping style in the pair as a whole, at least one member would frequently exhibit active coping, as seen in 37% of our cases. Although the direct impact on treatment adherence was not an objective of this study, it remains an interesting area for future research.

Our second objective was to explore the factors associated with shared passive coping styles, as well as compensatory or nonshared coping styles. For shared passive coping, the most relevant factor was multiple hospitalizations, possibly indicative of an unfavorable therapeutic response. Although our cross-sectional design does not permit establishing causality, it underscores the importance of evaluating coping strategies in patients with poor response, as well as the potential lack of support in family networks that may predispose individuals to passive coping. Furthermore, the absence of diagnostic information for the adolescent predisposed them to passive coping, reinforcing the recommendation to inform adolescents of their diagnoses so they can better understand and face their condition.

In cases where the adolescent coped actively while the caregiver was passive, we identified 2 main adolescent factors (younger age and not knowing the diagnosis) and 1 caregiver factor (psychiatric symptoms) that limited the possibility of the adolescent’s active coping reversing the caregiver’s passive stance. Although these adolescents might cooperate with treatment, they could still be hindered by a lack of emotional support from the caregiver. Hence, the involvement of a multidisciplinary health team, including mental health professionals, is essential in these scenarios.

Conversely, when passive coping was observed in the adolescent, the caregiver often remained active, possibly motivated by perceiving a good prognosis. This attitude could lead to a more cooperative environment and better mood, especially if the caregiver feels compelled to be strong for the adolescent and other children in the family. However, factors like fewer support networks can increase the caregiver’s personal burden. For adolescents in this group, not knowing their diagnosis can predispose them to a passive coping style by limiting their ability to process or confront the reality of the disease.

Regarding time of progression of cancer, our results do not suggest a significant influence on coping styles, which stands in contrast to the findings of Bruce E. Compas, where patients with progression of 6 months to 1 year exhibited poorer adaptation [28]. This discrepancy could suggest that other elements, such as communication patterns or psychological interventions provided early in the treatment, may moderate the effect of disease duration.

Regarding the confounding variables analyzed—patient or caregiver gender, education level, religion, residence outside Mexico City, the adolescent’s interest in the diagnosis, family function, and the patient’s psychiatric diagnosis—none showed a significant effect on coping styles.

### Study limitations and future directions

Our study has the following limitations. First, its cross-sectional design precludes causal inference and prevents us from tracking how coping evolves over time [29]. Second, all data were gathered in a single Mexican tertiary centre, which may limit generalisability to other cultural or clinical settings. Third, coping and psychosocial variables were self-reported, introducing potential reporting bias. Fourth, we did not measure resilience, personality or other dispositional constructs that can shape coping [6, 30, 31]. Fifth, although the sample size was adequate for the primary analyses, it may have lacked power to detect smaller effects.

Despite these limitations, the study retains notable strengths. It used instruments validated for Spanish-speaking populations and administered by personnel trained in paediatric psycho-oncology. Moreover, the observation that passive coping in one member does not automatically translate into passive coping in the other underscores the caregiver–adolescent dyad as a distinct therapeutic unit, reinforcing the rationale for systemic rather than purely individual interventions.

Future longitudinal or interventional studies, ideally multicentre, should follow coping trajectories from diagnosis through each treatment phase to clarify how styles develop and influence long-term adaptation and adherence.They should also compare systemic, dyad-based interventions aimed at strengthening coping in both members with individual programmes that target only the member who shows coping difficulties.

Overall, our findings highlight the value of routinely assessing coping styles in both adolescents and their primary caregiver to guide psychosocial care, improve collaboration with the treatment team and ultimately enhance well-being in paediatric oncology contexts.

## Conclusions

In contexts where adolescents face cancer, primary caregivers most often display an active coping style. When the caregiver’s coping is passive, the adolescent was observed to compensate with an active style in just over half of the dyads. Our findings suggest that the caregiver–adolescent dyad should be viewed as a single therapeutic unit, reinforcing the rationale for family-centred systemic interventions aimed at strengthening coping in both members.

## Data Availability

All data analysed are included in the article; the underlying datasets are not publicly available but can be obtained from the corresponding author on reasonable request. No supplementary information files are provided.

## Abbreviations

AC: Adolescent with cancer
ACS: Adolescent Coping Scale
APGAR: Adaptability, Partnership, Growth, Affection, and Resolve
CI: Confidence interval
CSI: Coping Strategies Inventory
OR: Odds ratio
PC: Primary caregiver
SD: Standard deviation

## References

[1] Bleyer A, Ferrari A, Whelan J, Barr RD. Global assessment of cancer incidence and survival in adolescents and young adults. Pediatr Blood Cancer. 2017;64:1–9. 10.1002/pbc.26497 28244694

[2] SINAVE/DGE/SALUD/Perfil epidemiológico de cáncer en niños y adolescentes en México. 2011. https://epidemiologiatlax.files.wordpress.com/2012/10/p_epi_de_los_tumores_malignos_mc3a9xico.pdf. Accessed 31 May 2016.

[3] Fajardo GA. Mortalidad por cáncer en niños. Bol Med Hosp Infant Mex. 2005;62:1–3.

[4] Wu WW, Tsai SY, Liang SY, Liu CY, Jou ST, Berry DL. The mediating role of resilience on quality of life and cancer symptom distress in adolescent patients with cancer. J Pediatr Oncol Nurs. 2015;32(5):304–13. 25612835 10.1177/1043454214563758

[5] Wen WW, Chang JT, Tsai SY, Lian SY. Assessing self-concept as a mediator between anger and resilience in adolescents with cancer in Taiwan. Cancer Nurs A. 2018;41(3):210–7. 10.1097/NCC.0000000000000512 28537955

[6] Haase JE, Kintner EK, Robb SL, Stump TE, Monahan PO, Phillips C, Stegenga KA, Burns DS. The resilience in illness model Part 2: confirmatory evaluation in adolescents and young adults with cancer. Cancer Nurs. 2017;40(6):454–63. 27984241 10.1097/NCC.0000000000000450 PMC5471133

[7] Turner-Sack AM, Menna R, Setchell SR, Maan C, Cataudella D. Psychological functioning, post-traumatic growth, and coping in parents and siblings of adolescent cancer survivors. Oncol Nurs Forum. 2016;43(1):48–56. 26679444 10.1188/16.ONF.48-56

[8] Kim DH, Chung NG, Lee S. The effect of perceived parental rearing behaviors on health-related quality of life in adolescents with leukemia. J Pediatr Oncol Nurs. 2015;32(5):295–303. 25631364 10.1177/1043454214563412

[9] Chen CM, Du BF, Ho CL, Ou WJ, Chang YC, Chen WC. Perceived stress, parent-adolescent/young adult communication, and family resilience among adolescents/young adults who have a parent with cancer in Taiwan. Cancer Nursing A. 2018;41(2):100–8. 10.1097/NCC.0000000000000488 28410333

[10] Lazarus R, Folkman S. Estrés y procesos cognitivos. España: Martínez Roca; 1986.

[11] Murphy LK, Bettis AH, Gruhn MA, Gerhardt CA, Vannatta K, Compas BE. Resilience in adolescents with cancer: association of coping with positive and negative affect. J Dev Behav Pediatr. 2017;38:646–53. 28816911 10.1097/DBP.0000000000000484

[12] Sanjari M, Heidari S, Shirazi F, Salemi S. Comparison of coping strategies in Iranian adolescents with cancer and their parents. Issues Compr Pediatr Nurs. 2008;31:185–97. 19021038 10.1080/01460860802475176

[13] Bradley E, Hallemeier AG, Fried TR, et al. Documentation of discussions about prognosis w Sanjariith terminally patients. Am J Med. 2011;111(3):218–23. 10.1016/s0002-9343(01)00798-7 11530033

[14] Compas BE, Desjardins L, Vannatta K, Saleme TY, Rodriguez EM, Dunn M, Bemis H, Snyder S, Gerhardt CA. Children and adolescents coping with cancer: self- and parent reports of coping and anxiety/depression. Health Psychology. 2014;33(8):853–861. 10.1037/hea0000083 PMC4241756 25068455

[15] Allen CE, Kelly KM, Bollard CM. Pediatric lymphomas and histiocytic disorders of childhood. Pediatr Clin North Am. 2015;62(1):139–65. 25435117 10.1016/j.pcl.2014.09.010 PMC4250829

[16] Cano García FJ, Rodríguez FL, García MJ. Adaptación española del Inventario de Estrategias de afrontamiento. Actas Esp Psiquiatr. 2007;35(1):29–39.17323223

[17] Smilkstein G. The family APGAR. A proposal for a family function test and its used for physicians. J Fam Pract. 1978;6:12–31. 660126

[18] Burgdorf J, Colechio EM, Stanton P, Panksepp J. Positive emotional learning induces resilience to depression: a role for NMDA receptor-mediated synaptic plasticity. Curr Neuropharmacol. 2017;15(1):3–10. 27102428 10.2174/1570159X14666160422110344 PMC5327454

[19] Bellón JA, Delgado A, Luna JD, Lardelli P. Validez y Fiabilidad del Cuestionario de Función Familiar Apgar- familiar. Aten Primaria. 1996;18(6):289–95. 8983381

[20] Peduzzi P, Concato J, Kemper E, Holford TR, Feinstein AR. A simulation study of the number of events per variable in logistic regression analysis. J Clin Epidemiol. 1996;49(12):1373–9. 8970487 10.1016/s0895-4356(96)00236-3

[21] Martínez-Montilla JM, Amador-Marín B, Guerra-Martín MD. Estrategias de afrontamiento familiar y repercusiones en la salud familiar: Una revisión de la literatura. Enferm Global. 2017;16(3):576.

[22] Gómez-Clavelina FJ, Irigoyen-Coria A, Ponce-Rosas ER. Selección y análisis de instrumentos para evaluación de la estructura y funcionalidad familiar. Arch Med Fam. 1999;1(2):45–57.

[23] Castillo MID, Juárez VLE, Palomo CMA, Medina SA, Zapata TM. Calidad de vida en niños con leucemia linfoblástica aguda durante la inducción a la remisión mediante el PedsQL Cancer Module. Bol Med Hosp Infant Mex. 2009;66:410–8.

[24] King A. Neurobiology: rise of resilience. Nature. 2016;531(7592):S18–9. 26934522 10.1038/531S18a

[25] Espinoza SNR, Zapata del Mar CM, Mejía Pérez LA. Conspiración de silencio: una barrera en la comunicación médico paciente y familia. Rev Neuropsiquiatr. 2017;80(2):125–36.

[26] Reuben JD, Shaw DS. Resilience in the offspring of depressed mothers: variation across risk, domains, and time. Clin Child Fam Psychol Rev. 2015;8(4):300–27. 10.1007/s10567-015-0195-5 26541559

[27] Costas MR. Hispanic adolescents coping with parental cancer. Support Care Cancer. 2012;20:413–7. 21953284 10.1007/s00520-011-1283-9 PMC4104649

[28] Compas BE, Jaser SS, Dunn MJ, Rodriguez EM. Coping with chronic illness in childhood and adolescence. Annu Rev Clin Psychol. 2012;8:455–80. 22224836 10.1146/annurev-clinpsy-032511-143108 PMC3319320

[29] Talavera JO, y Rivas RR. Del juicio clínico a la encuesta transversal. Rev Med Inst Mex Seguro Soc. 2012;50(6):641–4. 23331750

[30] Miller KS, Vannatta K, Compas BE, Vasey M, McGoron KD, Salley CG, Gerhardt CA. The role of coping and temperament in the adjustment of children with cancer. J Pediatr Psychol. 2009;34(10):1135–43. 19451171 10.1093/jpepsy/jsp037 PMC2782254

[31] Harper FWK, Goodlett BD, Trentacosta CJ, Albrecht TL, Taub JW, Phipps S, Penner LA. Temperament, personality, and quality of life in pediatric cancer patients. J Pediatr Psychol. 2014;39(4):459–68. 24443742 10.1093/jpepsy/jst141 PMC3994318

